# Supplementary material for: Scalable dissolution-dynamic nuclear polarization with rapid transfer of a polarized solid
Source: Nat Commun. 2019 Apr 15;10:1733. doi: 10.1038/s41467-019-09726-5 (PMC6465283; doi:10.1038/s41467-019-09726-5)
Supplement: Supplementary file 1 — Supplementary Information [file 41467_2019_9726_MOESM1_ESM.pdf]

# Scalable Dissolution-Dynamic Nuclear Polarization with Rapid Transfer of a Polarized Solid

Karel Kouřil,* Hana Kouřilová, Samuel Bartram, Malcolm H. Levitt, and Benno Meier†

*School of Chemistry, University of Southampton,*

*Southampton, SO17 1BJ, United Kingdom*

(Dated: March 7, 2019)

**SUPPLEMENTARY FIGURES**

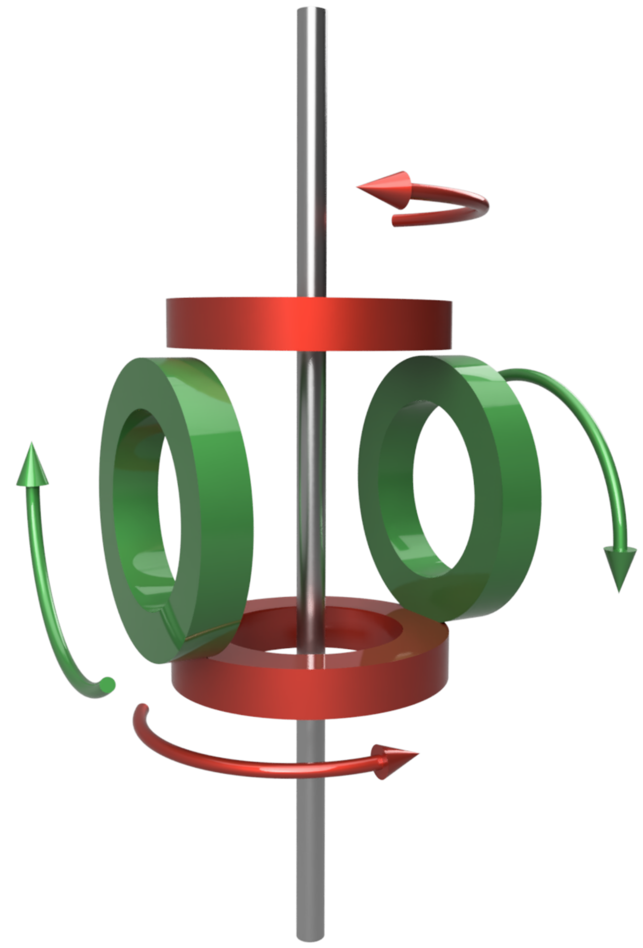

Supplementary Figure 1. Helmholtz-Anti-Helmholtz coil arrangement used to rotate the magnetic field when the sample is transferred. The sample travels through the stainless steel tube in the centre. A first Helmholtz coil pair (green) creates a magnetic field oriented perpendicular to the sample direction of travel. The fields generated by the coils of a second, Anti-Helmholtz coil pair (red) oppose each other: the lower coil generates a field along the sample travel direction while the upper coil generates a field which is antiparallel to the direction of travel. The diameter of each coil is approximately 6 cm. Each coil is made of approximately 100 turns of 1 mm diameter copper wire. The arrows indicate directions of electric currents in the coils.

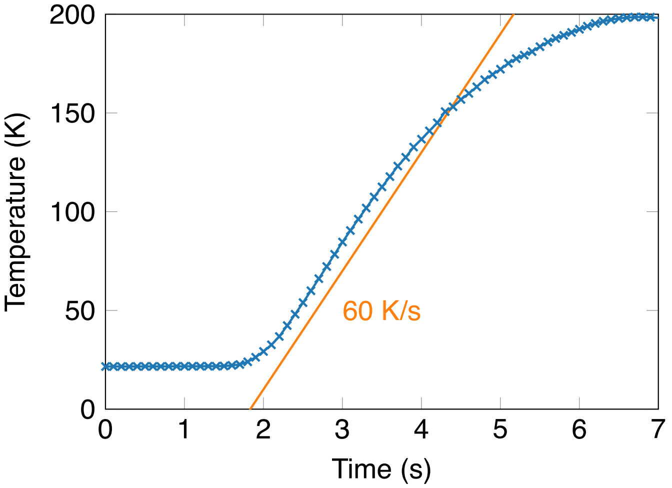


Supplementary Figure 2. Time-dependence of the sample temperature in flowing helium gas. The sample is equilibrated in a liquid helium dewar at approximately 20 K. The sample is then rapidly moved to the top of the dewar where it comes to a rest in a tube with rapidly flowing, ambient temperature helium gas. The straight line is a guide to the eye with a gradient of 60 K/s.

## SUPPLEMENTARY METHODS

### The DNP insert

A combination of standard RG-174 coaxial cable (RS Components Ltd, UK) and stainless steel coaxial cable (Aspen Electronics, UK) is used to connect the tuned and matched RF coil to an N-port on the top of the insert. The helium-level is measured using Allen-Bradley resistors and a home-built controller. The temperature inside the cryostat is measured using a Cernox sensor connected to an Oxford Instruments temperature controller. The top of the insert uses a KF50 6-way cross (Kurt J Lesker Ltd, UK) to connect the vacuum pump to the sample space and to connect the RF, Allen-Bradley and temperature cabling as well as fibres for the optical detection to the outside. A 188 GHz, 50 mW microwave source (ELVA-1, Sweden) is connected to the top of the microwave tube.

### The transfer tunnel

The flexible transfer tunnel is built by tightly winding 1 mm diameter copper wire (RS) onto a 6 mm OD nylon tube (RS). The windings are held in position with insulating tape.

### System control

The microwave source is controlled from the computer via a serial interface. The Allen-Bradley resistors are excited and measured using a custom circuit, that interfaces to an Arduino microcontroller. A second Arduino controls a series of mechanical relays that switch 24V lines that in turn control pilot valves (SY3A00-5U1, SMC) that switch the various valves and the pneumatic actuator on top of the injection device. This Arduino also controls the temperature of the solvent reservoir of the injector. All Arduinos are controlled via ethernet. All control software on the computer is written in Python, software running on the Arduinos is written in C++.

A programmed routine on the main computer sends a command to the main power supply to energize the coils. After 200 ms a further command is send to the Arduino to start the sample ejection, dissolution and injection routine and to trigger the NMR acquisition.

**Sample Heating**

A PT-1000 Temperature Sensor (IST INNOVATIVE SENSOR TECHNOLOGY, P1K0.161.6W.A.010) was calibrated between 6 K and 290 K using a Cernox CX-1010-SD-HT-0.1M temperature sensor as follows: Both sensors were mounted on a home-built probe. The probe was inserted into a liquid helium dewar. In order to calibrate the sensor the probe was slowly pulled out of the dewar. The temperature of the calibrated sensor and the resistance of the PT-1000 sensor were recorded approximately every 1 K. A Mercury temperature controller (Oxford Instruments) was used to log the data.

The actual temperature measurements were carried out using the calibrated sensor and a Keithley 2400 source meter. A current of 0.3 mA was used for callibration and to record the resistance. The source meter was interfaced to a computer via serial interface, and temperature values were recorded every 100 ms.

The setup consisted of two parts: (i) A straight 1/4" steel tube plugged at the bottom and with a provision to flow helium gas through the top 10 cm of the tube. This tube was inserted into a helium dewar. (ii) A probe made of 1/8" PEEK tube with the PT-1000 sensor attached to the bottom end, which can be slid into the 1/4" tube. A bullet with pyruvic acid was attached to the bottom of the probe so that the sensor was submerged in the acid. The sample with the sensor was then frozen in liquid nitrogen, and the probe was inserted into the dewar through the 1/4" tube and left to equilibriate. The temperature recording was started, and the probe was rapidly lifted to the top section of the tube where it was exposed to the rapidly flowing ambient temperature helium gas.
